# Chlorhexidine is not an essential component in alcohol-based surgical hand preparation: a comparative study of two handrubs based on a modified EN 12791 test protocol

**DOI:** 10.1186/s13756-017-0258-0

**Published:** 2017-09-13

**Authors:** Thomas-Jörg Hennig, Sebastian Werner, Kathrin Naujox, Andreas Arndt

**Affiliations:** 1 B. Braun Medical AG, Centre of Excellence Infection Control, Seesatz 17, 6204 Sempach, Switzerland; 2 HygCen Germany GmbH, Bornhövedstrasse 78, 19055 Schwerin, Germany

**Keywords:** Surgical hand antisepsis, Surgical scrubbing, Chlorhexidine, Alcohol, Handrub evaluation

## Abstract

**Background:**

Surgical hand preparation is an essential part of modern surgery. Both alcohol-based and antiseptic detergent-based hand preparation are recommended practices, with a trend towards use of alcohol based handrubs. However, discussion has arisen whether chlorhexidine is a required ingredient in highly efficacious alcohol-based formulations, in view of providing sustained antimicrobial efficacy.

**Methods:**

One alcohol-only formulation (product A), containing ethanol and n-propanol, and one formulation containing a chlorhexidine-ethanol combination (product B) were directly compared with each other using a modified test protocol based on European standard EN 12791 (2016) with 25 volunteers. The alcohol-only formulation (product A) was applied for only 90 s, the chlorhexidine-alcohol formulation (product B) for 180 s. Microbial log reduction factors were determined and statistically compared immediately after application and at 6 h under surgical gloves.

**Results:**

The alcohol-only formulation (product A) achieved mean log reduction factors of 1.96 ± 1.06 immediately after application and 1.67 ± 0.71 after 6 h. The chlorhexidine-alcohol combination (product B) achieved mean log reduction factors of 1.42 ± 0.79 and 1.24 ± 0.90 immediately and after 6 h, respectively. The values for product A were significantly greater than those for product B at both measured time points (*p* ≤ 0.025 immediately after application and *p* ≤ 0.01 after 6 h).

**Conclusions:**

An optimized alcohol-only formulation tested according to a modified EN 12791 protocol in 25 healthy volunteers outperformed a chlorhexidine-alcohol formulation both immediately after application and at 6 h under surgical gloves, despite a much shorter application time. Thus, optimized alcohol-only formulations do not require chlorhexidine to achieve potent immediate and sustained efficacy. In conclusion, chlorhexidine is not an essential component for alcohol-based surgical hand preparation.

## Background

Surgical hand preparation, also termed surgical hand antisepsis or surgical scrubbing, has become an essential part of modern surgery. It was introduced as part of the post-Listerian system of aseptic surgery that was widely adopted in Europe and the USA at the turn of the twentieth century [1]. The goal of surgical hand preparation is to generate a near-elimination of transient hand flora or hand contamination, and a substantial reduction of resident hand flora that would be sustained for the duration of the surgery [2]. The aim is to prevent wound contamination or infection by accidental glove leaks or glove rupture. Although the basic necessity of surgical hand preparation, as compared to no preparation, has never been tested in a randomized clinical trial (RCT), the practice is nevertheless strongly supported by the principles of microbiology, by existing models of pathogen transmission and by empirical observations. Hands are frequently contaminated with microorganisms, accidental sterile glove leaks are common and there are observations of case clusters of surgical infections when hand preparation protocols were inadequate or breached [2–5].

The two main approaches to surgical hand preparation are (a) aqueous preparation, using antiseptics such as chlorhexidine (CHG) or povidone-iodine (PVI) in a detergent base that are applied and then rinsed off with running water, and (b) preparation with alcohol-based handrubs, without rinsing with water, where the alcohol provides the bulk of the microbicidal action. The preference for either of the two approaches differs in different healthcare settings, and both aqueous and alcohol-based hand preparation have been incorporated as suitable alternatives into important major guidelines [2, 6, 7]. Alcohol-based hand preparation has four main advantages over aqueous preparation, (a) it generally achieves much greater reduction of microorganisms on hands, (b) formulations with suitable emollients are usually associated with less skin damage and dermatitis, (c) it requires shorter application times, and (d) it saves considerable amounts of running water [2, 3, 8, 9]. One large RCT of alcohol-based versus aqueous preparation found equivalence in terms of surgical site infection (SSI) rates and concluded that the alcohol-based protocol was better tolerated by the surgical teams and improved compliance with hand hygiene guidelines [10]. Modern alcohol-based handrub formulations can meet efficacy requirements within application times as short as 1.5 min, which can translate to time savings for surgeons and surgical teams [11].

The efficacy of surgical hand preparation formulations and protocols is routinely tested using standardized in vivo microbiological protocols on the hands of volunteers [2]. The European standard EN 12791 [12] compares a given formulation to a reference alcohol consisting of 60% (*v*/v) n-propanol on clean hands and stipulates that the microbial log reduction factors of the tested formulation must not be statistically inferior to the reference, both immediately after application and after 3 h under surgical gloves. The US standard ASTM E1115 [13], applied with the US Food and Drug Administration (FDA) criteria [14], stipulates that a given formulation on clean hands must achieve absolute microbial log reduction factors of 1.0 on the first day of application, of 2.0 on the second day, and of 3.0 on the fifth day after a total of 11 sequential applications, and must show sustained efficacy by way of microbial counts not exceeding those at baseline after 6 h under surgical gloves. Both standards require that adequately-validated neutralizers are used during testing, in order to prevent false-positive results from sustained antimicrobial activity that is exerted after the end of the planned antiseptic exposure [15].

Discussion has arisen as to whether alcohol-based surgical handrubs should contain CHG as a second agent, to ensure persistency of the antiseptic effects for the duration of the operation. A few studies [16, 17] indeed reported superiority of CHG-alcohol combination rubs over alcohol-only products at time points of up to 6 h under surgical gloves. However, concerns were subsequently raised about methodological details of these studies, such as whether initial antiseptic application and subsequent neutralization were adequate [18, 19]. In order to investigate this question further, we initiated a comparative study of an alcohol-only versus a CHG-alcohol combination handrub, based on a modified EN 12791 test protocol with an extended period of 6 h under surgical gloves.

## Methods

Two commercially available alcohol-based handrubs were investigated. Product A was an alcohol-only handrub containing 45% (*w*/*v*) ethanol, 18% (w/v) n-propanol and emollients (Softa-Man®, also branded as Softalind®, B. Braun, Sempach, Switzerland). Product B was a CHG-alcohol combination handrub containing 1% (*w*/w) chlorhexidine gluconate, 61% (w/w) ethanol and emollients (3 M™ Avagard™ Surgical and Healthcare Personnel Hand Antiseptic with Moisturizers, 3 M Health Care, St. Paul, MN, USA).

Twenty-five healthy volunteers were included in two experimental runs in a randomized, cross-over design according to a modified EN 12791 protocol [12]. The two modifications were that instead of using 60% (*v*/v) n-propanol as a reference, the two products (A and B) were directly compared as part of a benchmarking exercise, and that a prolonged period of 6 h instead of 3 h under surgical gloves was used to assess sustained efficacy. Hands were prewashed for 1 min with non-antiseptic soap. Pretreatment bacterial counts (prevalues) were obtained by rubbing fingertips and thumb tips in 10 ml tryptic soy broth (TSB) for 1 min. Subsequently, each subject used either product A or product B by applying 3 portions of 3 ml of the product to the hands, in such a way as to keep the hands moist with the product for the duration of the application. Product A was applied for a shortened application time of 90 s, product B was applied for the full 3 min duration, as usually done for the reference alcohol in EN 12791. Immediate postexposure bacterial counts (first postvalues) were taken from one hand by rubbing fingertips and thumb tips in 10 ml TSB containing neutralizers, and the other hand remained gloved for 6 h. Another set of bacterial counts (second postvalues) was taken after glove removal. Validated neutralizers (a combination of 5.0% polysorbate 80 + 0.6% sodium oleate +2.0% lecithine) according to EN 13727 [20] were used in the TSB for sampling (postvalues). Samples in TSB were plated on neutralizer-free tryptic soy agar (TSA), incubated at 36+/−1 °C for 48 h, and colonies were subsequently enumerated. The differences between the log_10_ pre- and postvalues (log reduction factors) were determined for each subject, and the means of these differences were statistically compared using the Wilcoxon matched-pairs signed-ranks test.

## Results

The results of the direct comparison between the alcohol-only (product A) versus the CHG-alcohol (product B) handrub are shown in Table 1. Product A achieved mean log reduction factors (± standard deviation) of 1.96 ± 1.06 immediately after application, and 1.67 ± 0.71 after 6 h. Product B achieved mean log reduction factors of 1.42 ± 0.79 and 1.24 ± 0.90 immediately and after 6 h, respectively. The mean log reduction factors obtained with use of product A were significantly greater than those of product B at both time points, immediately after application (*p* ≤ 0.025) and after 6 h under surgical gloves (*p* ≤ 0.01).

**Table 1:** Comparison of an alcohol-only handrub (product A) versus a chlorhexidine-alcohol combination handrub (product B) for surgical hand preparation in a modified test arrangement according to EN 12791 (2016)

				Immediate effects			6-h effects	
	Application time	Application volume	Prevalues(log_10_ ± SD)	Mean RF(log_10_ ± SD)	*p* value^a^	Prevalues(log_10_ ± SD)	Mean RF(log_10_ ± SD)	*p* value^a^
Product A (alcohol only)	90 s	3 × 3 ml	4.68 ± 0.5	1.96 ± 1.06	≤0.025	4.79 ± 0.51	1.67 ± 0.71	≤0.01
Product B (CHG-alcohol)	180 s	3 × 3 ml	4.79 ± 0.52	1.42 ± 0.79		4.73 ± 0.50	1.24 ± 0.90	

*RF* reduction factor, *SD* standard deviation, *CHG* chlorhexidine

^a^Calculated using the Wilcoxon matched-pairs signed-ranks test

## Discussion

Our results show that an alcohol-only handrub that contains an optimized composition of two alcohol species, ethanol and n-propanol, achieved a significantly greater initial microbial reduction after surgical hand preparation than a CHG-alcohol combination handrub when tested according to a modified EN 12791 protocol, and that a significant difference in reduction factors in favor of the alcohol-only rub was maintained for 6 h under surgical gloves. These results are consistent with findings from a previous comparative study [21], in which an 80% (*w*/w) ethanol-only rub passed EN 12791 requirements both immediately and at 3 h after application, while the same 1% (w/w) CHG and 61% (w/w) ethanol combination rub as was used in the present study failed at both time points. Among the three common alcohol species (ethanol, isopropanol and n-propanol) that are used for hand and skin antisepsis, n-propanol has the most potent general microbicidal activity and exerts this at relatively lower concentrations [22]. This supports its inclusion into alcohol-based handrubs and explains the potent activity exhibited by a combination of n-propanol and ethanol at an overall concentration of 63% (*w*/*v*) in product A as observed in the present study.

The results in this study were obtained despite the absence of CHG in handrub A, despite the extended test interval of 6 h under surgical gloves and despite the fact that handrub B was given a very substantial a priori advantage by having had twice the initial application time (180 s) of handrub A (90 s), with the application volume (3 × 3 ml) and other conditions being equal. These findings are consistent with earlier findings indicating that the application of alcohols, despite the absence of residual activity per se, is followed by substantially delayed regrowth of skin flora or even continued microbial killing [23, 24] and that the contribution of dedicated supplements for persistency is relatively modest or even absent when tested for durations of up to 6 h [25, 26]. This is further consistent with the concept that an initially strong and immediate microbial killing capacity continues to translate into low viable microorganism numbers for several hours under surgical gloves. The extended duration of 6 h under surgical gloves was chosen for the present experiments, instead of the usual 3 h for EN 12791, in order to simulate tougher test conditions that resemble those of the US ASTM E1115 standard in this aspect. Furthermore, our results as well as those of others [11, 27] support shorter application times, as short as 1.5–2 min, when highly potent alcohol-based handrubs are used.

In addition to using an effective alcohol-based handrub, the technique of application is also considered important [28]. While EN 12791 requires coverage of only the hand surfaces for the purpose of efficacy testing, the World Health Organization hand hygiene guidelines [2] state that complete coverage of hands and forearms requires about 15 ml (about 3 × 5 ml) and emphasize that it is important to keep hands and forearms wet with alcohol for the entire duration of application. Some protocols that include very small applied volumes [29] are therefore a cause for concern, as they are likely to lead to incomplete skin surface coverage and interrupted action of the antiseptic.

In the area of skin antisepsis, CHG has been the subject of a considerable amount of incorrect assessment. What had happened was that a substantial proportion of the clinical trial-based literature on skin antisepsis had attributed favorable clinical outcomes achieved with CHG-alcohol combinations (two effective antiseptics) to CHG alone and concluded that CHG alone (instead of the combination) was the agent supported by evidence [30, 31]. This had happened despite a strong microbiological literature base that showed alcohols to be potent antiseptics. The misinterpretation of trial outcomes was carried over into prominent guidelines, led to a number of prominent recommendations for CHG alone, and created widespread but in parts unsubstantiated views of CHG as an “in” antiseptic.

Microbiological assessment of antiseptics may similarly be subject to errors. Both European and US standards stipulate that adequately validated neutralizer substances must be used at the point of sampling after antiseptic exposure [15]; this is in order to prevent continued killing due to residual bacteriostatic or bactericidal action after the end of the dedicated exposure. While this applies to the testing of all antiseptics, CHG in particular is prone to false-positive efficacy assessment in the absence of adequate neutralization, due to strong bacteriostatic activity that it exerts at concentrations far below bactericidal levels [32–34]. It has been suggested that this is a factor that likely led to a systematic skewing of the antiseptic literature [35]. Previous reports of superior performance of CHG-alcohol combination rubs for surgical hand antisepsis [16, 17] indeed attracted letters to the editor that expressed concern about adequate neutralization [18, 19]. In the present study, adequate neutralizers were used during the entire testing process, thus creating equal sampling conditions for both comparator rubs.

Among the different hand hygiene agents, alcohol-based handrubs are generally most well tolerated on skin; true alcohol allergies have not been documented beyond reasonable doubt, and irritant contact dermatitis from alcohols is rare when handrubs are well formulated with emollients [2]. On the other hand, CHG is a known allergen and a frequent cause of irritant contact dermatitis among healthcare personnel [2, 36, 37]. It has also been the subject of a recent US FDA warning about rare but serious anaphylactic reactions [38]. Thus, there is a clear potential for a tolerability advantage from alcohol-only handrubs for surgical hand preparation, especially if their antimicrobial performance characteristics are equal to or even better than those of CHG-containing ones.

It is a limitation of our study that it was performed according to a modified EN 12791 protocol, and results from EN 12791 testing do not necessarily translate into congruent results according to the US standard ASTM E1115 [13], mainly due to the US standard’s requirement for incremental increases (cumulative effects) in log reduction factors on subsequent days of testing [15, 39]. However, the clinical relevance of this requirement has been questioned, because it is not intuitive why a surgeon’s hands after antisepsis should be permitted to have different microbial counts on different working days of the week [39]. In any case, EN 12791 is a very stringent standard in which only very potent handrubs tend to pass, and it has high inter-laboratory reproducibility [40].

## Conclusions

In the present study, an alcohol-only handrub containing a mix of ethanol and n-propanol outperformed a CHG-alcohol handrub both immediately after application and at 6 h when applied for 1.5 min and tested according to a modified EN 12791 protocol in 25 healthy volunteers. This means that optimized and well-formulated alcohol-only handrubs can provide superior performance to CHG-alcohol combination rubs for the purpose of surgical hand preparation, even with shortened application times, and this includes sustained efficacy for an extended period of 6 h under surgical gloves. In conclusion, given that CHG has a greater potential for skin irritation than alcohols alone, CHG is not an essential or even necessary component of alcohol-based handrubs for surgical hand preparation. Further studies in routine practice are warranted.
